# A Zebrafish Embryo as an Animal Model for the Treatment of Hyperpigmentation in Cosmetic Dermatology Medicine

**DOI:** 10.3390/medicina54030035

**Published:** 2018-05-25

**Authors:** Ahmad Firdaus B. Lajis

**Affiliations:** 1 Department of Bioprocess Technology, Faculty of Biotechnology and Biomolecular Sciences, University Putra Malaysia, 43400 UPM Serdang, Selangor, Malaysia; 2 Laboratory of Molecular Medicine, Institute of Bioscience, University Putra Malaysia, 43400 UPM Serdang, Selangor, Malaysia; 3 Bioprocessing and Biomanufacturing Research Center, Faculty of Biotechnology and Biomolecular Sciences, University Putra Malaysia, 43400 UPM Serdang, Selangor, Malaysia

**Keywords:** bioactive agent, danio rerio, melanin, melanogenesis, pigmentation, tyrosinase

## Abstract

For years, clinical studies involving human volunteers and several known pre-clinical in vivo models (i.e., mice, guinea pigs) have demonstrated their reliability in evaluating the effectiveness of a number of depigmenting agents. Although these models have great advantages, they also suffer from several drawbacks, especially involving ethical issues regarding experimentation. At present, a new depigmenting model using zebrafish has been proposed and demonstrated. The application of this model for screening and studying the depigmenting activity of many bioactive compounds has been given great attention in genetics, medicinal chemistry and even the cosmetic industry. Depigmenting studies using this model have been recognized as noteworthy approaches to investigating the antimelanogenic activity of bioactive compounds in vivo. This article details the current knowledge of zebrafish pigmentation and its reliability as a model for the screening and development of depigmenting agents. Several methods to quantify the antimelanogenic activity of bioactive compounds in this model, such as phenotype-based screening, melanin content, tyrosinase inhibitory activity, other related proteins and transcription genes, are reviewed. Depigmenting activity of several bioactive compounds which have been reported towards this model are compared in terms of their molecular structure and possible mode of actions. This includes patented materials with regard to the application of zebrafish as a depigmenting model, in order to give an insight of its intellectual value. At the end of this article, some limitations are highlighted and several recommendations are suggested for improvement of future studies.

## 1. Introduction

Melanin, a pigment secreted by melanocytes in the basal layer of the epidermis, serves to protect human skin from ultraviolet radiation, free radicals and reactive oxygen species [1]. Accumulation of pigment in the skin causes pigmentation disorders, such as melasma, freckles, solar lentigo, and post-inflammatory hyperpigmentation [1,2]. For years, clinical trials using human volunteers and several known preclinical trials using in vivo models (i.e., mice, guinea pigs) have demonstrated their reliability in evaluating the effectiveness of a number of depigmenting agents [3,4,5,6,7,8]. Although these models have great advantages, they also suffer from several drawbacks especially related to ethics, animal welfare and humane endpoints in animal experimental units. All research involving in vivo models oblige to follow standard ethic committee guidelines, where the “3 Rs” principle should be fully implemented (i.e., Replacement, Refinement and Reduction). In accordance with these principles, zebrafish as a new depigmenting model has been proposed [9,10,11,12]. The application of this lower vertebrate has gained attention in the medicinal and cosmetic industry. At present, studies have demonstrated its novelty and reliability for evaluating many depigmenting agents [3,6,13,14,15]. Previous and very recent evidence in genomics, molecular genetics, genetic development and molecular biology on zebrafish pigmentation showed its correlation to human pigmentation [16,17,18,19,20,21]. For instance, the pigmentation gene SLC24A5 (NCKX5) homologous to that in zebrafish golden mutants appears to have an ortholog highly similar in sequence and more functionally significant in the evolution of depigmentation in the ancestors of modern Europeans because of its effect on diminished melanosome size, number and density during melanogenesis [19]. Polymorphism of the pigmentation gene SLC24A5 is also associated with the darker skin of African ancestors. In addition, the SLC45A2 gene encodes a Membrane-Associated-Transporter-Protein which regulates melanosomal pH and melanogenic enzyme activity as demonstrated in the zebrafish model [22]. Moreover, melanogenesis in zebrafish is comparable to that of human melanogenesis, which enables functionality study and protein interaction. For instance, the zebrafish model has been used to demonstrate the functionality of endoplasmic reticulum (ER) calcium sensor protein STIM1 (stromal-interaction-molecule-1) domain in regulating melanogenesis via interaction with cell membrane-localized adenylyl cyclase 6 (ADCY6) [23]. Adenylyl cyclise is coupled to melanocortin (MC) receptors where hormones such as α-melanocyte stimulating hormone (α-MSH) binds. In comparison, mammals have five MC receptors (MC1R-MC5R) and one or two melanocyte-concentrating hormone (MCH) receptors while zebrafish have six MC receptors, including two MC5R orthologues and three MCH receptors [1,10,24,25]. The function of cyclic adenosine monophosphate (cAMP) in zebrafish melanogenesis is most likely different or has more than one function as compared to that in mammals, where the former act as an intermediary for pigment translocation and finding intact microtubules [26]. These processes are equally important for both melanin dispersion and aggregation [26].

**Figure 1:**
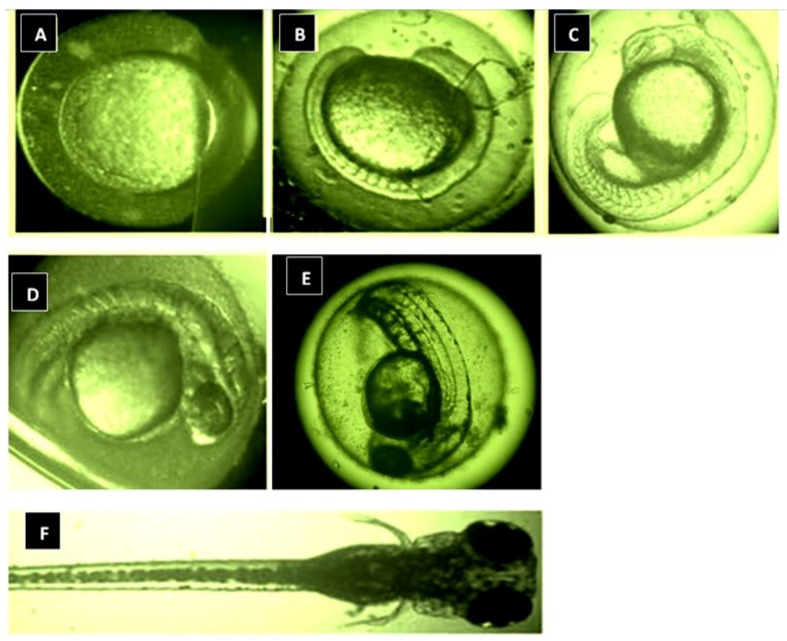
Zebrafish embryogenesis. Blastula at 0 h-post fertilization (hpf) (**A**), Embryo at 12 hpf (**B**), Embryo at 24 hpf (**C**), Embryo at 36 hpf (**D**), Larvae at 48 hpf (**E**), Larvae at 72 to 144 hpf (**F**). Note: hpf, hour-post fertilization.

The zebrafish pigment pattern originates from the neural crest (NeC)-derived stem cells to generate melanophores (melanocytes), xanthophores and iridophores via intermediate progenitors [10,21,27,28,29]. Recent studies have shown that these progenitors are multipotent without fate restriction, in order to give rise to three distinctive adult pigment cell types during embryogenesis to metamorphosis [30]. Typical zebrafish embryogenesis is illustrated in Figure 1. Several pigment-specific genes and proteins such as SOX9 and SOX10 are not only present in mouse but also in zebrafish, influencing the differentiation of NeC during its generation that is essentially important as developmental regulators of melanogenesis [31,32,33]. For instance, SOX10 controls the transcription of microphthalmia-associated transcription factor (MITF), a regulator for the expression of melanogenesis-related enzymes, including tyrosinase (TYR), tyrosinase-related proteins 2 (TRP-2), pigment cell-specific pre-melanosomal protein (PMEL) and tyrosinase-related proteins 1 (TRP-1) [29,30,34]. SOX10 also acts as a transactivator protein in the expression of TRP-2 genes required for melanogenesis, in which MITF cannot self-activate [30,35]. On the other hand, PMEL is necessary for pre-melanosomal fibril formation and melanosomal shape where its defect in PMEL-mutant zebrafish is suspected to cause loss in melanocyte viability [30]. Recent studies have also shown that other regulatory proteins including Wnt signaling remain active in differentiating melanophores and contribute to elevated transcription of MITFa, melanophore specification, and morphology in Zebrafish embryos [36]. Other similarities shared by zebrafish and mammalian models is that membrane protein of the zebrafish embryo chorion is identified to be homologous to proteins ZP2 and ZP2 of mouse zona pellucida [30,37,38].

This new model serves as a reliable model and tool to study various depigmenting agents. The present article discusses several methods that have been employed using this model to date, such as phenotype-based screening, melanin content, TYR inhibitory activity, other related proteins and transcription gene assays. The applications of this model towards several known and newly discovered depigmenting agents are also compared and demonstrated. Evidence highlighting the relationship between in vitro models and the molecular structure of bioactive compounds are illustrated to explain their possible interaction and effectiveness on zebrafish depigmentation. Some discussion on patents related to the zebrafish model as a depigmenting model are rarely reported in the literature but still contain valuable information regarding its intellectual property, thus have been included in this review. At the end of this article, some limitations regarding this model are discussed and several recommendations are suggested for improvement of further studies.

## 2. Protocol and Assays

### 2.1. General Procedures

In brief, several important parameters are considered in depigmenting assays and experimentation such as zebrafish strains, embryonic age, medium, temperature, inducers and experimental duration. Most of the time, wild-type (WT) zebrafish was chosen as compared to other transgenic variant or mutants where the experiment was initiated at embryonic stage at 2–12 h post fertilization (hpf) [39,40,41]. During depigmenting assays, the embryo was commonly incubated in an egg-water medium at an ambient temperature (25–30 °C) and pH ~7 [39,42]. Controlled temperature is one of the important parameters during the process from husbandry to an assay which may affect the overall zebrafish depigmenting analysis. For instance, it was found that the pigmentation of zebrafish melanophore stripes was reduced at a very low temperature milieu (i.e., 17 °C), as compared to that of zebrafish at 26.5 °C due to downregulation of gene expression levels for TYR and TRP-2, during melanogenesis [9,35]. Observation on zebrafish skin (at 17 °C) via optical and high-resolution transmission electron microscopy (TEM) showed a significant decrease in the number of melanophores, without affecting melanosomal aggregation [9]. On the other hand, a very acidic or basic pH can significantly reduce the amount of eggs and their survival rate in egg-water [39,42].

### 2.2. Effect of Phenylthiourea and Stimulating Hormones

Meanwhile, no additional nutrient was added at earlier embryonic age as the embryo obtained its nutrient from its yolk. Prior to any addition of bioactive compounds, the endogenous pigment is removed using phenylthiourea (PTU), an organosulfur TYR inhibitor regularly used to block pigmentation in zebrafish via inhibiting TYR-dependent melanogenesis pathway without any adverse toxicity [15,43,44]. It was suggested that PTU at a concentration of 75 µM effectively reduced pigmentation in the zebrafish, without considerably affecting mortality or exerting any teratogenicity effect [31]. Other than being a TYR inhibitor, recent studies demonstrated that PTU also contributed to zebrafish depigmentation via its anti-thyroidal effect [45]. In this particular, it was suggested that thyroid hormones regulate zebrafish melanin synthesis in a gender-dependent manner [45]. On the other hand, it has been demonstrated that pigmentation in zebrafish embryos could be stimulated via α-MSH but little is known about the effect of other stimulating hormones and compounds (i.e., isobutylmethylxanthine, 8-methoxypsoralen, forskolin, adrenocorticotropic hormone, 3,4-dihydroxyphenylalanine, stem cell factor) [11,40,46]. Nevertheless, a hormone such as MCH significantly reduced melanin dispersion and aggregation in zebrafish embryos [24,26,47]. The MC1R in zebrafish allows stimulation of melanogenesis in the presence of α-MSH due to fact that MC1R has the highest affinity for α-MSH [26]. In mammals, this is then stimulating melanogenesis via the G protein-coupled receptor (GPCR)-cAMP-MITF pathway to up-regulate TYR, TRP-1 and TRP-2 [25,48]. In zebrafish, it has been established that MC1R mediates melanosomal dispersal as demonstrated using knockdown MC1R expression via morpholino oligonucleotides [26].

### 2.3. Other Consideration

The effect of light on zebrafish embryos has been demonstrated and needs to be considered. Embryos at the age of 48 hpf showed an increased melanosomal dispersion when illuminated to visible light, whereby its body pigmentation increases on a bright background [49,50]. The light-induced melanosomal dispersion in zebrafish embryos may serve to protect it from the impact of ultraviolet (UV) irradiation [49,50]. This recent discovery of the effect of light on zebrafish embryos could be used as a new approach to investigate the capability of depigmenting agents to inhibit pigmentation in zebrafish embryos under UV light. In addition, depigmenting assay was mainly conducted at an early age of zebrafish embryos (24–72 hpf). This is due to the fact that within 14–24 hpf, different skin layers representing the epidermis and dermis can be recognized, although the cutaneous basement membrane zone at the dermal-epidermal junction is not yet developed. Hence, at this early age of a zebrafish embryos, small biomolecules or compounds can passively diffuse or percutaneously be absorbed via skin (i.e., epidermis and dermis) when oral structure has not been fully developed. However, as it reaches maturation age (168 hpf), many organs have been fully developed and it starts to absorb small compounds orally via different delivery routes which may give other implication on the effect of bioactive compounds to the embryo [51]. Moreover, at an age of more than 72 hpf, exposure to light will also induce an initial-rapid melanosomal dispersion, followed by a slow aggregation that will eventually lead to a pale body color [49,50]. These effects may probably give false results or lead to interference in depigmenting analysis.

### 2.4. Phenotypic Evaluation, Melanin Content and TYR Assays

**Figure 2:**
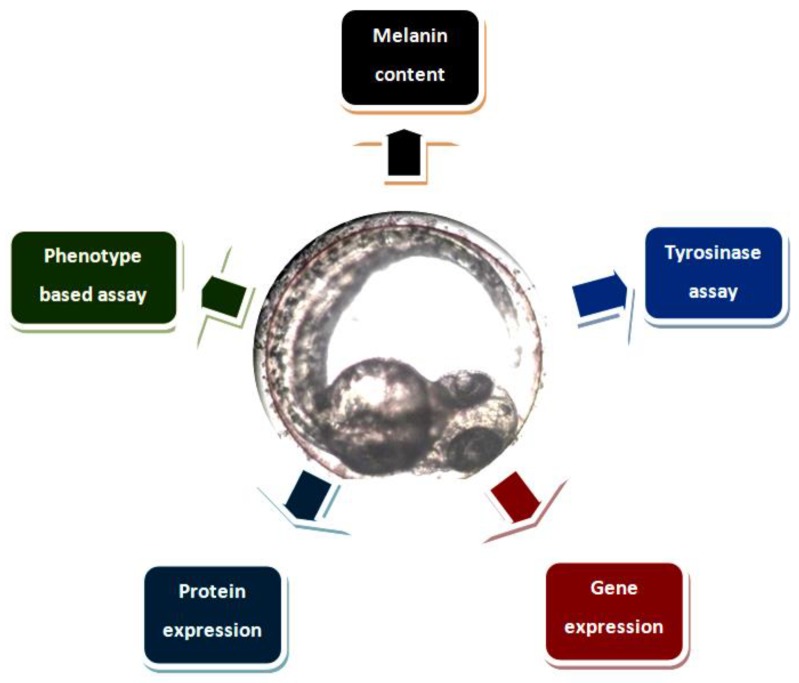
Depigmenting assays using zebrafish embryo model.

Figure 2 exemplifies several known depigmenting assays using the zebrafish embryo model. For phenotype-based observation, embryos were dechorionated using forceps, anesthetized in tricaine mesylate (C_10_H_15_NO_5_S) solution, and mounted in 3% (*w*/*v*) methyl cellulose [42,51,52]. The body pigmentation of zebrafish (i.e., dorsal and lateral) was visualized using a stereomicroscope and quantified by software (i.e., Image pro-plus (Media Cybernetics Inc., Rockville, MD, USA)) [6]. Other microscopic analysis includes TEM and scanning electron microscopy (SEM). For melanin content assay, zebrafish was commonly dissolved in NaOH, an alkaline solution with a combination of high temperature while for TYR assay. Zebrafish was sonicated, harvested and incubated with precursors (i.e., tyrosine, L-3,4-dihydroxyphenyl)alanine (L-DOPA)) which later quantified using absorption spectroscopy (AS) at respective wavelength [39,42]. NaOH at 1 M is enough to cause plasma membrane imbalance, breakdown and degrading of biomolecules which allow the solubilization of melanin for quantification. Alternatively, Soluene-350 is also used to solubilize melanin in cell or tissue samples for melanin quantification possibly suitable for zebrafish embryo melanin quantification. Other methods includes oxidation of melanin via alkaline H_2_O_2_ which later produces specific markers of black melanin (i.e., pyrrole-2,3,5-tricarboxylic acid and pyrrole-2,3-dicarboxylic acid) and measured using high-performance liquid chromatography (HPLC). In stark contrast to TYR assay, zebrafish cells were disrupted via sonication at a certain amplitude and frequency at low temperature to allow TYR protein release. The TYR activity quantification was only done after 7 hpf due to the fact that the TYR gene transcription and TYR activity was only detected at as early as 3 to 7 hpf before visible pigmentation in the retinal pigment epithelium (RPE) layer and then later followed by whole body melanin-pigmentation (approximately after 24 hpf) [10,35].

### 2.5. Protein and Gene Expression Assays

For protein expression assays (i.e., TYR, TRP-1, TRP-2, MITF), proteins were separated with sodium dodecyl sulfate-polyacrylamide gel electrophoresis (SDS-PAGE), blotted onto membranes (i.e., polyvinylidene fluoride), saturated with non-fat dried milk mixtures, exposed to specific primary antibodies and followed by incubation with a horseradish peroxidase (HRP)-conjugated secondary antibody to allow blot development i.e., via enhanced chemiluminescence detection system) [53]. The bands were physically observed or alternatively measured via densitometric analysis using software (i.e., Image MasterTM 2D Elite, G:BOX Chemi, LAS-1000 lumino-image analyzer) [53,54,55]. For gene expression assay, analysis was performed via qRT-PCR where total RNA was extracted and detailed protocol related to temperature, time, chemicals (i.e., oligo (dT), reverse transcriptase, oligonucleotides primers for TYR and glyceraldehyde-3-phosphate dehydrogenase (GAPDH) as an internal standard) and cycles have been previously described [53,56]. TRP-2 is an early marker for NeC-derived melanocytes (melanophores) and other melanin-synthesizing cells in the RPE as compared to TYR and TRP-1, which were expressed a bit later [35]. In WT zebrafish embryos, expression of TRP-1 paralogs (i.e., TRP-1a and TRP-1b) overlaps in the RPE and in melanocytes (melanophores) [35,55]. Alteration of amino acid “ARG” to amino acid “CYS” in the amino-terminal part of the TRP-1a in mutant zebrafish is similar to mutations in humans which lead to blond hair in Melanesians [55]. These are the most general and largest number of assays that have been implemented so far although many other assays could be performed in the future.

## 3. Application of Zebrafish Model

The evaluation of antimelanogenic activity of several depigmenting agents using zebrafish embryos has been increasingly reported. The application of zebrafish depigmenting assay was supported by the effect of well-known bioactive agents such as kojic acid and arbutin on embryo depigmenting activity [40,57,58]. Connections with many previous and current data of kojic acid and arbutin depigmenting assay in vitro (i.e., melanocytes) and in vivo (i.e., mice) help to support current investigation and better understanding using zebrafish embryo [13,57]. For instance, compound bis(4-hydroxybenzyl)sulfide reduces pigmentation level to about 41% (a percentage relative to untreated control), lower than arbutin and kojic acid, which indicates that bis (4-hydroxybenzyl)sulfide had a better depigmenting activity as compared to that of arbutin and kojic acid [13]. In addition, it was revealed that the bis(4-hydroxybenzyl)sulfide reduces melanogenesis in zebrafish comparably as effective as indicated by reduced melanin content, TYR, TRP-1, MITF in melanocytes in vitro [6,13]. These findings have gained enormous interest from the cosmetics industry to develop the most powerful and safe depigmenting agents [59].

### 3.1. The Effect of Small Molecular Weight Compounds on Zebrafish Depigmentation

Bioactive compounds subjected to zebrafish embryo depigmenting assay can be divided into several categories and molecular size. In general, the increasing mass number of the atoms or the chain length can increase the molecular weight and overall size of the molecules. Table 1 shows bioactive compounds of lower molecular weight (100–300 g/mol) and their depigmenting activity towards zebrafish embryo. For instance, gallic acid strongly reduced pigmentation of zebrafish embryo to about 40% (percentage in relative to untreated control) at concentration of 40 µM, comparably similar to bis(4-hydroxybenzyl)sulfide, but had higher depigmenting activity than kojic acid and arbutin [6,13]. On the other hand, raspberry ketone reduced melanin content and TYR activity in zebrafish embryo to about 60% (percentage in relative to untreated control) at 600 µM comparable to kojic acid [60]. On the contrary, ascorbic acid had no significant reduction of melanin content and TYR activity relative to untreated control [61]. Glabridin, a reversible noncompetitive TYR inhibitor as demonstrated via mushroom tyrosinase assay, also had no significant inhibitory effects on melanin synthesis in zebrafish [62]. In contrast, sesamol, a bioactive lignan of *Sesamum indicum*, concentration-dependently inhibited melanin biosynthesis in zebrafish embryo. The decrease of zebrafish pigment formation by sesamol can be explained by reduced TYR activity and melanogenesis-related gene expression [63]. Results from zebrafish depigmenting assay were in agreement with a decreased level of TYR, TRP-1, TRP-2, MITF activity, cAMP and MC1R in melan-a cells [63]. The p38 mitogen-activated protein kinase (p38 MAPK) and c-Jun N-terminal kinase (JNK) were also some major proteins involved in melanogenesis pathways. Thus, further evaluation explained that sesamol inhibited melanogenesis in melan-a cells via p38 MAPK and JNK pathway [63]. On the other hand, β-Lapachone of *Tabebuia avellanedae* at concentration of 0.8 μM remarkably inhibited melanin synthesis and TYR activity in zebrafish embryo which led to its pale phenotypic body pigmentation [64]. The depigmentation in zebrafish embryos can be further explained by reduced expression of TYR and TRP-1 at transcriptional gene and translational protein levels in melan-a cells [64]. The decreased level of MITF was coupled with delayed activation of extracellular signal-regulated kinase (ERK) by β-lapachone treatment [64,65]. Activation and acceleration of the degradation of ERK has a significant effect on MITF expression [54,65]. Furthermore, β-lapachone reduced melanogenesis in the reconstituted 3D human skin tissue culture, MelanoDermTM (MEL-300-B, MatTek Corporation, Ashland, USA) as indicated by brightness value (L*) within 2–3 weeks [64].

### 3.2. The Effect of Intermediate Molecular Weight Compounds on Zebrafish Depigmentation

The depigmenting activities of bioactive compounds having molecular weight of 300–500 g/mol are presented in Table 2. The depigmenting activity of Biochanin-A seen in zebrafish depigmenting assay was related to in vitro cell line and mice dermal depigmenting assays although the concentration and time may vary [3]. Biochanin-A reduced zebrafish embryo pigmentation in a dose-dependent manner. It inhibited 50% pigmentation relative to untreated control at concentration of 176 µM. This is consistent with the reduction of melanin content and cellular TYR activity in B16 cells [3]. Moreover, Biochanin-A (2%, *w*/*w*) cream formulation applied on mice skin in twice-daily basis significantly increased the skin-lightening index (L* value) within 2 weeks [3]. In comparison, omeprazole reduced pigment area density in zebrafish embryo to 63% (by 37% inhibition) at low concentration (60 µM). In addition, intracellular TYR activity was also decreased by 48% (relative to untreated zebrafish embryo) upon omeprazole treatment [53]. Moreover, molecular analysis via qRT-PCR confirmed the reduction TYR, TRP-1a, TRP-2 and MITFb mRNA expression levels in a concentration-dependent manner upon omeprazole treatment [53,56]. MITF is well-known for its important role in the development of melanocytes and melanin. It is worth noting that the zebrafish genome contains two MITF (i.e., MITFa and MITFb), in lieu of one MITF in the mammalian model [70]. NeC-derived melanophores require MITFa for differentiation and are absent in nacre/MITFa zebrafish mutants [30,66]. Zebrafish mutants for the MITF ortholog MITFa show a complete absence of body pigmentation and melanophores [70]. Zebrafish genomes also contain two TRP-1 paralogs (i.e., TRP-1a and TRP-1b) [55]. Knockdown of both TRP-1a and TRP-1b results in the formation of brown pigments instead of black eumelanin coupled by severe melanosome defects in zebrafish embryos [55]. Therefore, it is worth demonstrating the effect of depigmenting compounds to all MITF and TRP-1 ortho/paralogs in order to understand better its mechanism of actions.

In zebrafish, other proteins such as Sox10 and Wnt signals are also involved as positive regulators of MITFa and a transcription factor directly drives melanophore cell fate via the MITFa promoter in multiple NeC lineages while Foxd3, a winged helix transcription factor, was suggested for being a negative regulator of melanophore development [36,80]. It has been demonstrated that the zebrafish embryo treated with glyceollin showed a significant reduction of in situ expression of Sox10, in the neural tubes of the trunk region of the embryo. Meanwhile, in vitro study showed that glyceollin inhibited stem cell factor (SCF)/c-KIT signaling pathways in B16 cells as well as significantly impaired expression and activity of MITF as analyzed via immunoblots analysis [76]. It has been previously demonstrated that SCF/c-KIT greatly influenced melanin pigmentation and effectively upregulated intracellular cAMP levels in mammalian cells [24,81]. Thus, evidence that showed the inhibition of glyceollin towards SCF/c-KIT signaling pathways, Akt phosphorylation and downregulation of cAMP levels in B16 cells may be a possible explanation of its mechanism of action in zebrafish embryo depigmentation [76]. In zebrafish, KIT signaling is also essentially required for development and survival of embryonic and early metamorphosis of melanophores progenitors as demonstrated from sparse/KITa and sparse-like/KIT ligand-a analysis [81].

In zebrafish, KITa and KITb are two orthologues of mammalian KIT and only KITa is expressed in the melanophore lineage [81]. In KITa homozygous null zebrafish mutant; the melanophores appear to differentiate normally but they were decreased in number by about 40%, less migration, and ultimately experience apoptosis in relative to WT control. A very recent study demonstrated the overlapping controls of other transcription factors (i.e., Transcription Factor Activator Protein 2 alpha (TFAP2a) and epsilon (TFAP2e)) on KITa expression level and melanophore characteristics (i.e., viability and differentiation) in zebrafish embryos [70,81]. In zebrafish TFAP2a homozygous null mutants, KITa expression was reduced and embryonic melanophores demonstrate limited migration [81,82]. On the other hand, TFAP2a/e embryonic double mutants showed small and under-melanized melanophores, even though it retains some MITFa expression level [70,82]. Forcing expression of MITFa in TFAP2a/e double mutants partially restores their melanophore differentiation [70,83].

On the other hand, Gomisin at a concentration of 30 µM reduced protein levels of TYR, TRP-1, TRP-2 and MITF in zebrafish embryos to about 40%, 80%, 80% and 80% respectively as quantified using Image MasterTM 2D Elite software for densitometric analysis of the bands. This is in relation to downregulation of MC1R, adenylyl cyclase 2, MITF, TYR, TRP-1, and TRP-2 in vitro. Moreover, Gomisin treated melan-a cells exhibit elevated p-Akt and p-ERK levels, which imply that melanogenesis inhibition was via the activation of the PI3K/Akt and MAPK/ERK pathways [54].

Also, it has been demonstrated that hydroxylated amide derivatives compound known as 6d had a high-potency inhibitory activity in zebrafish embryo melanogenesis as compared to kojic acid, which was mainly due to the formation of irreversible complexes with the target TYR enzyme [52]. This is supported by the fact that compound 6d also inhibited TYR activity in A375 cells by 91% in relative to untreated control at a concentration of 50 µg/mL [52]. As compared to compound 6d, the Haginin-A is a non-competitive inhibitor that exhibits relatively strong depigmenting activity in the zebrafish model and decreased its intracellular TYR activity [52,73]. Other compounds such as oleoylethanolamide reduced the body pigmentation in the zebrafish model to about 49.5% (in relative to untreated control) at concentration of 150 µM [78]. This is supported by the in vitro study where Haginin-A and oleoylethanolamide substantially downregulated MITF, TYR, and TRP-1 protein expression via induction of ERK and Akt/PKB in a concentration-dependent manner in melan-a cells and B16 cells respectively [77,78]. Haginin A also decreased UV-induced the skin pigmentation in vivo model using brown guinea pigs [77]. Arctigenin also reduced pigmentation in zebrafish embryos, correlated with reduction of melanin content and TYR activity on B16 and melan-a cells [58]. In vitro analysis showed that arctigenin had better depigmenting activity as compared to kojic acid and arbutin and modulate melanogenesis via decreasing the cAMP level and promoted the phosphorylation of ERK (p-ERK) [58]. Interestingly, evidence shows that arctigenin modulates melanogenesis (i.e., lowers TYR, increases p-ERK expression) by both dose and time-dependent manner, which describes its cellular pharmacodynamic behavior and efficiency [58].

### 3.3. The Effect of High Molecular Weight Compounds on Zebrafish Depigmentation

Table 3 shows the antimelanogenic activity of bioactive compounds of molecular weight between 500–1000 g/mol. For instance, Floralginsenoside-A (MW of 786 g/mol) at concentration of 80 mM reduced melanin content and TYR activity in zebrafish embryo to about 80–84% (relative to untreated control) [42]. Antimelanogenic activity of Floralginsenoside-A was comparatively similar to that of Ginsenoside-Rb2 [39,42]. The 4,5-O-Dicaffeoylquinic Acid inhibited pigmentation in the zebrafish embryo in a dose dependent manner [31]. At the highest concentration tested (25 µM) of 4,5-O-Dicaffeoylquinic Acid, depigmentation level in zebrafish embryos was reduced to about 30% (relative to untreated control) whereby significant shrinkage of the melanocytes in the head region of the embryos was observed. This is correlated with partial inhibition of TYR activity in zebrafish, which led to decreasing melanogenesis activity in various body parts, including the head region of the embryos [31]. Other high molecular weight compounds such as Octaphlorethol-A also reduced melanin content and TYR activity in zebrafish embryos in a dose-dependent manner [84]. Most high molecular weight bioactive compounds significantly inhibited melanogenesis in zebrafish embryos only at high concentration as compared to small molecular weight molecules. Moreover, intermolecular forces (i.e., London dispersion, van der Waals forces) of high molecular weight molecules are high, which later influences their viscosity and dispersion in solution.

### 3.4. The Effect of Crude Extract on Zebrafish Depigmentation

Another crude extract and formulation containing several bioactive compounds was also reported in several studies (Table 4). For instance, *Salix alba* extract at concentration of 400 ug/mL reduced melanin content in zebrafish embryos to about 40% (relative to untreated control) [85]. This is due to the presence of high phenolic content and depigmenting compounds, majorly lupeol, 3,3′-di-O-methyl ellagic acid and hydrolysable tannins [85]. These depigmenting compounds were known to inhibit TYR activity in vitro [85]. Other crude extracts such as *Anoectochilus* extract inhibited the production of melanin in zebrafish embryos [56,86]. The mRNAs of melanin-related genes, such as PMEL, TYR, TRP-1a, were downregulated by the *Anoectochilus* extracts temporally and spatially in zebrafish embryos [56]. The *Anoectochilus* extracts also inhibited TYR enzymatic activity in a concentration-dependent manner [56]. Herbal prescription LASAP-C containing several plant extracts such as *Angelicae Dahuricae Radix*, *Rehmanniae Radix Crudus*, *Lycii Fructus*, and *Scutellariae Radix* showed a remarkable decrease in zebrafish embryo pigmentation [87]. *Ganoderma formosanum* mycelium extract (400 ppm) and *Blumea balsamifera* L. flavonoid (300 ug/mL) reduced melanin content to about 50% and 42% respectively, relative to untreated control [44,66]. Other extracts from marine *Pseudomonas*, *Anoectochilus* and *Narcissus* had a potent effect on zebrafish embryo depigmentation [56,86,88,89].

**Figure 3:**
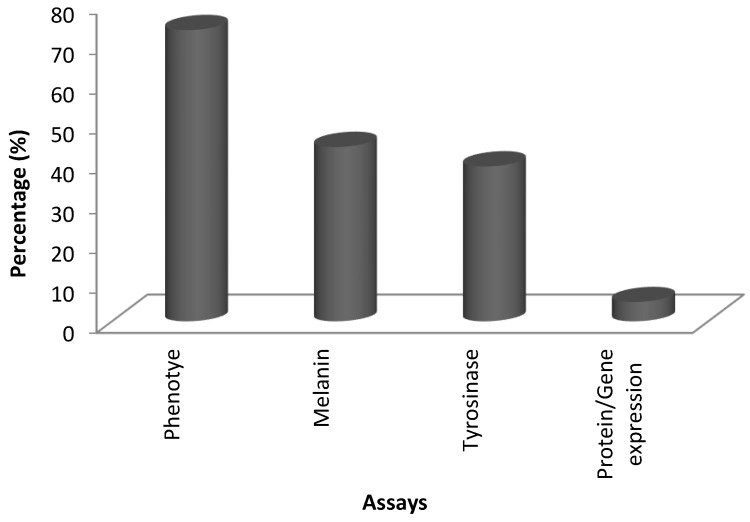
Statistical analysis of number of studies for type of assays.

By using the National Center for Biotechnology Information Search database (NCBI), Espacenet and a combination of other bioinformatics and search tools available, about 51 bioactive compounds, crude extract and formulations have been subjected to zebrafish embryo depigmenting analysis to date as presented in Table 1, Table 2, Table 3 and Table 4. Out of these, about 70% has been studied using phenotype-based analysis, 44% assessment on their effect on melanin content, 39% measured via TYR inhibition assay and only about 5% have been studied in detail via molecular work on gene-protein expression (Figure 3). Phenotype-based evaluation provides rapid evaluation on depigmenting activity of bioactive compounds due to ease of observing zebrafish as compared to in vitro and other in vivo models. Melanin content and TYR assays are relatively easy to implement while protein-gene expression is rarely conducted probably due to limited availability of markers, proteins, antibodies and molecular instruments for studies.

The zebrafish embryo model in depigmenting assay has been regarded as a valuable intellectual property. This technology has been patented by several institutions or companies covering various aspects and areas of interest [41,46,71,92,93]. For instance, zebrafish embryos have been used as a method to evaluate safety and their effectiveness of various depigmenting agents via statistical zebrafish mortality and phenotypic pigmentation [92]. Other patents relate to the application of suloctidil in the preparation of skin-lightening products [79]. The depigmenting activity of suloctidil was proven via its inhibition potential against melanin and tyrosinase in zebrafish embryos [79]. Other institutions patented on the application of transgenic zebrafish TG (KIT: RAS) embryo, which exhibit overproliferation of melanophores as early as from 48 hpf [79,81]. In this recent technology, a melanoma transgenic zebrafish was generated by means of the GAL4-UAS system to overexpress RAS in melanophores [81]. Using the combinatorial Gal4-UAS system, a zebrafish transgenic line that expresses oncogenic HRAS under the KITa promoter was developed [81]. At about 72 hpf, KITa-GFP-RAS transgenic mutants show a hyper-pigmenting phenotype as the earliest evidence of abnormal melanophore growth. In a study, compound 2-methylphenyl-E-(3-hydroxy-5-methoxy)-styryl ether substantially inhibited the melanophore overproliferation in transgenic embryos, within 24–48 hpf relative to untreated control [41,81]. Also, the application of other depigmenting compounds including tretinoin, kojic acid, gallic acid, MEK-I, 2-Morpholinobutyl)-4-thiophenol, arbutin, niacinamide, and Haginin-A have been described in patents using zebrafish embryo [41,77].

**Figure 4:**
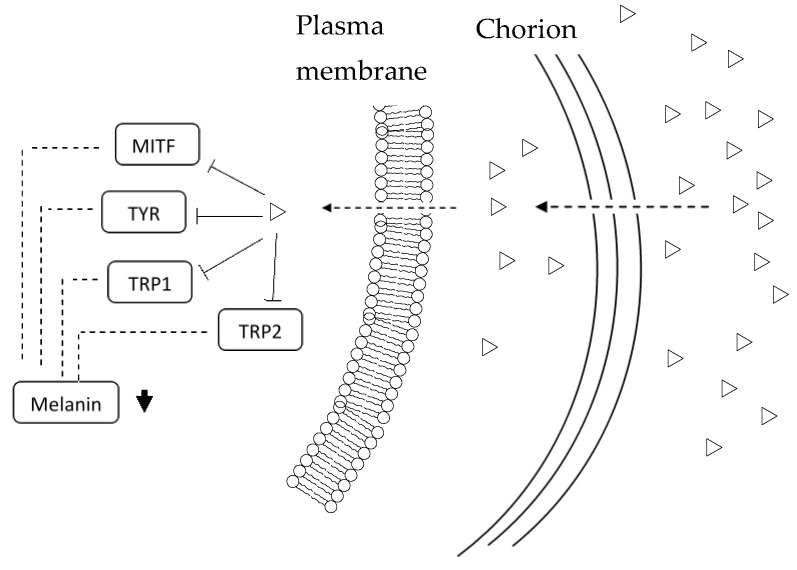
Proposed schematic diagram explains possible mechanism of action of depigmenting agents on zebrafish embryo depigmentation. Zebrafish embryo chorion had a specific nanoporosity on the external membrane (500–700 µM in diameter) [37,38]. Chorion, organized as a three-layered structure (i.e., extraembryonic mesoderm), with four major polypeptides (i.e., N-linked glycoproteins) [37,95,96]. Moreover, hydrophobic molecules and at slow rate very small uncharged polar molecules can diffuse via lipid bilayer. The passive diffusion rate through a membrane is proportional to the LogP of the molecules between the membrane (lipophilic milieu) and the external medium (aqueous environment). Denotes symbols: 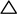, depigmenting agent; 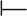, inhibition; 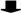, downregulation.

Most of these compounds share a similarity in the way that they had a benzene ring structure with varied number of hydroxyl groups (OH) attached to it. These attributes may tend their biological activity towards reduction of pigmentation, melanin and TYR activity in zebrafish embryos. Apparently, their molecular size, stability, hydrophilicity and hydrophobicity are also different, which possibly contributes to their penetrability, passive membrane permeability and bioavailability in the process of embryo depigmentation. Hydrophobicity is needed for the compounds to permeate via the various biological membranes. In the case of the zebrafish embryos, several biological membranes should be considered including chorion, melanocytes cell membrane and melanosome plasma membrane [37,38]. The chorion with pore channel of around 0.5 to 0.7 μm diameter with gap at 1.5 to 2.5 μm intervals, which surrounds the embryo reduces the diffusion rate of small molecules into the embryo [37] (Figure 4). Hydrophobicity affects molecule absorption, bioavailability, hydrophobic molecules-receptor interactions, molecule metabolism and their toxicological endpoints [90,94]. LogP is a measure of molecular hydrophobicity, an important parameter in designing molecules and to determining its quantitative structure-activity relationship (QSAR) [94]. In particular, with a similar partition coefficient (LogP), the permeability of a smaller molecule is relatively higher than that of a larger molecule, thus allowing a number of smaller molecules to bind to TYR and inhibit its activity as compared to larger molecules. For instance, glyceollin (MW, 338.35 g/mol; logP, ~2.53) at concentration of 10 µM and Haginin-A (MW, 300.31 g/mol; logP, 2.45) at 4 µM inhibited TYR at about 60% and 55% respectively [76,77]. Hydrophobicity is a key factor in studies of the environmental fate and degradation of molecules. It is also a crucial determinant of the pharmacokinetic behavior of molecules, which influences their distribution into tissues, the binding characteristics of molecules and governing passive membrane partitioning. In particular, kinetic studies and docking stimulation indicated that compound 6d (logP, ~4) had better depigmenting activity than kojic acid (logP, −2) due to competitive inhibition of the oxidation of L-DOPA and formed irreversible complexes with the target enzyme TYR [52]. Lipophilic kojic acid derivative also showed better depigmenting activity than kojic acid in vitro and in vivo [40]. LogP is also an important factor in determining the solubility of bioactive compound [94]. Therefore, in a depigmenting assay, a suitable medium is necessary to solubilize bioactive compounds and act as carrier and vehicle for respective bioactive compounds. In particular, the use of dimethyl sulfoxide, a universal solvent at low concentration (0.1%, v/v), has been reported suitable and non-toxic for study [40,53].

## 4. Limitations

Although the zebrafish and mammalian models share similarities, there are also differences which limit application. The most obvious drawback is the physiology property of zebrafish embryos that differs to mammalian models. In particular, the mammalian epidermis has a true stratified epithelium next to a basement membrane that divides the epidermis from dermis [29]. The main divergence between zebrafish and human skin is that this fish model lacks mammalian appendages (i.e., sebaceous glands and hair follicles) [97]. The way that depigmenting compounds are absorbed into zebrafish embryos is different from that into mammalian skin. It has been reported that chorion pore canal has a viscosity of more than 200 times higher than egg-water, and limits the diffusion of even nanoparticles [37,38]. A very small molecule can diffuse slowly via the chorion, especially at a high concentration. This explains why the concentration and duration time for depigmentation process differs from these two models. These differences have greater implications for the pharmacodynamic and pharmacokinetic interpretation of depigmenting agents on different models. Moreover, the interpretation and correlation of the effect of depigmenting agents on these two models are still lacking. In addition, other interpretations of the side effect of depigmenting agents such as toxicity in zebrafish embryos to irritancy in human skin is still unknown [98]. Information on bioavailability and permeability of depigmenting agents using zebrafish embryos is also scarcely available. Ironically, the zebrafish embryo depigmenting assay is not a standalone model in which must be supported by in vitro and in vivo models. Studies on melanogenesis and mechanisms of action of various depigmenting agents are well documented using in vitro models, allowing study of its action at various level of melanogenesis including genes and proteins levels. Although modulation of the depigmenting agent at protein-gene expression level (i.e., TYR, TRP-1a, TRP-1, MITFb, Sox10) in the zebrafish embryo has been reported, and the number of studies regarding these approaches is still low [55]. Other protein (i.e., TRP-1 and MITF) ortho/paralogs are also not included in some protein-gene expression assays. In particular, for melanogenesis in mammalian melanocytes, the melanogenic enzymes are transcribed from DNA to mRNA which are later translated into proteins (i.e., TYR). Upon TYR translation and its introduction into the endoplasmic reticulum (ER), it is subjected to some initial glycosylation and maturation which later enters the Golgi, where it is transported to the melanosomes, by a vesicular transport system. This melanogenic enzyme resides in the melanosome plasma membrane and TRP2 is probably complexed with TYR and TRP-1 [34,69,99]. It can be only assumed that this typical process is also present during melanogenesis in zebrafish melanophores.

Other processes related to melanogenic pathways, melanosome formation and maturation in melanocytes (melanophore) of zebrafish embryo have not fully elucidated. Zebrafish melanocyte contains only eumelanin and a lack of pheomelanin, while in mammalian melanocyte, the melanosomes harbor two types of melanin (i.e., eumelanin and pheomelanin), which can separately be identified via various methods (i.e., chemical treatment followed by HPLC analysis, spectrophotometric method at ratio A650/A500, Raman spectroscopy, fluorimetric method, in vivo coherent Raman imaging) [100,101]. The synthesis of eumelanin in zebrafish melanocyte most likely follows the typical pathway of the hydroxylation of tyrosine to dihydroxyphenylalanine (DOPA) and later to DOPAquinone enzymatically catalyzed by TYR. DOPAquinone is converted into DOPAchrome that serves as a substrate for TRP-2 to catalyze the formation of 5,6 dihydroxyindole-2-carboxilic acid (DHICA). TRP-1 mediates the last step of melanogenesis by oxidizing DHICA to melanin [20]. In this particular pathway and due to the absence of pheomelanin, it is possible that the conversion of DOPAquinone to cysteinylDOPA and 5-hydroxy-1,4-benzothiazinylalanine (HBTA) does not exist in zebrafish melanocyte. Understanding of the relevant melanogenesis pathways in zebrafish embryos could be used to predict the mechanisms of action of depigmenting agents in zebrafish embryo pigmentation. For instance, melanogenesis in mammalian cells is also induced by the effect of free radicals and inflammatory agents, which this not fully studied in zebrafish embryo. Many studies focus on the effect of depigmenting compounds on melanocyte (black pigment) of zebrafish embryo. Studies on other types of pigment cells such as iridophores (pigment containing guanine) and xanthophores (pigment containing pteridine) is still lacking.

## 5. Recommendations

Although studies using zebrafish embryos have generated considerable data to demonstrate the depigmenting activity of various bioactive compounds, they are by no means comprehensive or complete. Recommendations for future studies include investigation of depigmenting compounds using embryos of other zebrafish variants (i.e., transgenic zebrafish with overproduction of melanin and zebrafish golden mutants which resemble pigmentation in the ancestors of modern Europeans) [30,81]. Other variants could possibly be used to mimic the actions of depigmenting compounds on different kinds of mammalian skin.

Furthermore, concerning depigmenting assays, other types of evaluation should be explored such as the effect of depigmenting agents on size, number and distribution of melanophore melanin in various parts of the body via TEM [20,21]. TEM Analysis could provide information on the average melanosomal density and maturity. Recently, it has been demonstrated that fluorescence spectroscopy method provides a more accurate and specific result for melanin quantification in zebrafish embryos as compared to typical AS method [100]. This is due to the capability of fluorescence spectroscopy method in distinguishing non-melanotic cells from those that are melanotic. [100]. Other mechanisms of action, such as the prevention of TYR glycosylation, hindrance of binding of cooper ions for TYR activation, inhibition on melanosome maturation, and melanosomal transportation to keratinocytes should also be explored to show possible diverse effects of depigmenting agents in zebrafish embryo depigmentation [97].

In the near future, studies on the interaction of melanocytes and keratinocytes could be possibly conducted using zebrafish embryos. Recent studies reported a well-demarcated keratinocyte structure with a surface contour consisting of microridges that can be observed via SEM in the developing skin surface of 24 hpf old zebrafish embryos, and they are very well organized by 144 hpf [97]. At least up to 144 hpf, the developing zebrafish epidermis has characteristics of marking features that can be changed by perturbed keratinocyte gene expression [97]. In mammalian cells, vasoactive peptides such as endothelins play major roles in pigmentation; for instance endothelin-1 (EDN1) of keratinocyte is a mediator of melanocyte dendricity and a new melanogen to direct expression of the TYR gene in UVB-exposed human epidermis [97,102]. In zebrafish, endothelins such as EDN3 are expressed in the zebrafish epidermis, probably by the keratinocytes surrounding the melanophores. The endothelins are known to act as key inducers of normal embryonic and mature melanophore formation by binding to the Endothelin Receptor Type B [102].

Instead of just being a model limited for depigmenting compound screening purposes, zebrafish embryos could be possibly used to study the effect of UV radiation, free radicals, oxidation and inflammatory-related hyperpigmentation [7,103,104]. In human skin, tissue damage and repair also frequently lead to alterations in skin pigmentation. Wounding of skin invites inflammatory cells to the affected area, which release cytokines that direct the activities of other cells including keratinocytes and melanocytes during the repair process [7,105]. Mechanisms of keratinocyte migration and melanocytes during the re-epithelialization phase of cutaneous injury healing in mammalian cells have been studied and well documented. A recent study also demonstrated the live-image of melanophores recruitment and their precursors, melanoblasts, to injury sites of zebrafish embryos. These pigment cells came after the inflammatory response which led to hyperpigmentation and scars [7,105]. This finding allows the molecular connection between inflammatory agents and pigment cells during tissue repair and also enables evaluation of depigmenting agents for wound hyperpigmentation treatments [1,2,7,106].

Recently, computational molecular dynamics (MD) and the umbrella sampling simulations model has been demonstrated as a comprehensive model for evaluating passive permeability of bioactive compounds via a lipid bilayer [72,94]. This computational model has been demonstrated as a functional, predictive tool for permeability prediction [68,94]. The results from the developed computational model has significant improved agreement and synergistic relationship with in vitro parallel artificial membrane permeability assay (PAMPA), relative to other existing methods (i.e., immobilized artificial membrane technique, and immobilized liposome chromatography) [52,90,94]. This new model could possibly be used to evaluate permeability of depigmenting compounds through zebrafish embryo membranes.

Replacing the mammalian model with the zebrafish embryo model for evaluation of efficacy and safety of depigmenting compounds is an interesting topic of discussion in the skin-care industry [73,74,75,92,107,108,109,110,111,112,113,114]. However, in the cosmetic or even dermatological industry, these compounds are formulated in combination of a carrier or vehicle to facilitate its effect on human skin [73,74,79,86,115]. So far, no studies or patents have been reported on the protocols and possibility of how such methods could be performed using zebrafish embryo models.

## 6. Skin Depigmentation: Pros and Cons

Regardless of its applications and benefits, skin depigmentation may also give adverse effect especially over long period of time. Melanin is a photoprotective component, and plays a physiological role as a UV absorbent, which is known to protect skin from harmful and excessive UV radiation intensity, DNA damage as well as development of cancer cells [1,16,114,116]. Melanin during melanogenesis, enzymes such as catalase, glutathione peroxidase and superoxide dismutase are synthesized and dedicated to removing radicals [i.e., reactive oxygen radical (ROS), superoxide anion (O_2_^•-^), hydrogen peroxide (H_2_O_2_), and singlet oxygen (^1^O_2_)] [1,16]. Modulation of melanogenesis may cause other health implications and problems. Long-term consequences of depigmentation agents in mammals and humans are normally related to hypersensitivity to UV light, development of contact dermatitis, leukoderma, redness and itching [104]. In comparison, these skin reactions were difficult or merely impossible to be seen using the zebrafish embryo model, especially to the naked eye [104,117]. Recent study showed that itch-stimulating pruritogens induce slightly different itch-like responses in the zebrafish and mammalian models [117]. The metabolisms and excretion in and out from the mammalian body is way too different from the fish model [72,106]. Moreover, cosmetic or medical formulation ingredients can be a complex mixture of several ingredients due to the presence of many components, which include steroids (i.e., fluocinolone acetonide and other corticosteroids) [5,20,79,110]. Such steroids aim to reduce sensitivity to cosmetic or medical formulation and skin discomforts. Those formulations also contain one or a mixture of several chemical (i.e., Avobenzone, oxybenzone) or physical (i.e., zinc oxide, titanium dioxide) sunscreen agents to absorb or block UV up to certain level (i.e., Sun Protection Factor (SPF)), depending on its amount and concentration. Even though some depigmenting compounds contain antioxidant properties, their formulation is normally accompanied by well-known antioxidants such as vitamin C and E. These components, together with depigmenting compounds, can effectively reduce melanogenesis in abnormal melanocytes in skin, protect against UV radiation and reduce the toxic effect to the normal melanocytes.

## 7. Conclusions

The zebrafish embryo is a powerful model, proven to be efficient in the evaluation of various depigmenting agents. Nevertheless, it also has some limitations and differences as compared to mammalian models. Known for its advantages, it facilitates and accelerates the screening and assessment process of various bioactive compounds. Recent progress, knowledge and evidence on zebrafish pigmentation, as well as its interaction with various internal and external factors, have allowed better evaluation and understanding of its mechanism of action. Therefore, this model is deemed to make a significant impact with regard to its contribution to the knowledge and development of cosmetic product formulation as well as to provide better alternative safer drugs for the pharmaceutical industry.

## Figures and Tables

**Table 1:** The bioactive compounds (low molecular weight, 100–300 g/mol) and their antimelanogenic activity.

Bioactive Compounds	Description	ICp	Icm	ICt	References
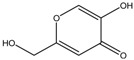 Kojic acid	Formula: C_6_H_6_O_4_ MW: 142.11 Origin: *Apergillus/Penicillium* Log P: −2.45	~90% (50 µM)	~80% (50 µM) ~50% (20 mM)	~60% (20 mM)	[13,44,52,66]
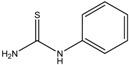 Phenylthiorea	Formula: C_7_H_8_N_2_S MW: 152.22 Origin: Synthetic Log P: 0.73	-	~55% (30 ug/mL) ~30% (200 ppm)	~45% (30 ug/mL) ~20% (200 ppm)	[43,44,67]
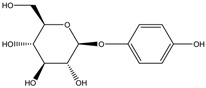 Arbutin	Formula: C_12_H_16_O_7_ MW: 272.25 Origin: Bearberry plant Log P: −0.58	~75% (10 mM)	-	-	[13]
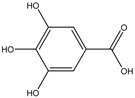 Gallic acid	Formula: C_7_H_6_O_5_ MW: 170.12 Origin: Plant Log P: 0.47	~40% (50 µM)	-	-	[6]
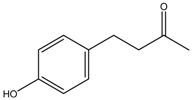 Raspberry ketone	Formula: C_10_H_12_O_2_ MW: 164.20 Origin: Raspberry Log P: 2.07	PO	~60% (600 µM)	~60% (600 µM)	[60]
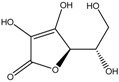 Ascorbic acid	Formula: C_6_H_8_O_6_ MW: 176.12 Origin: Plant Log P: −3.36	-	No effect (0.5 mM)	No effect (0.5 mM)	[61]
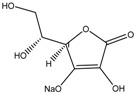 Sodium erythorbate	Formula: C_7_H_10_NaO_6_ MW: 213.14 Origin: Synthetic Log P:-	-	~60% (300 mM)	~60% (300 mM)	[61]
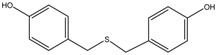 Bis(4-hydroxybenzyl)sulfide	Formula: C_14_H_14_O_2_S MW: 246.32 Origin: *Gastrodia elata* Log P: 3.50	~50% (53 µM)	-	-	[13]
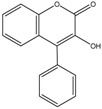 4-phenyl hydroxycoumarins	Formula: C_15_H_10_O_3_ MW: 238.24 Origin: Synthetic Log P: 2.04	~50% (5–25 ug/mL)	-	-	[68]
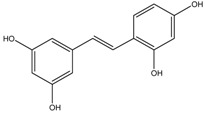 Oxyresveratrol	Formula: C_14_H_12_O_4_ MW: 244.24 Origin: *Morus alba* Wood Log P: 2.67	PO	-	-	[51]
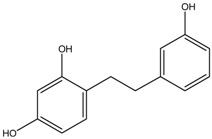 2,4,3′-Trihydroxydihydrostilbene	Formula: C_14_H_14_O_3_ MW: 230.26 Origin: *Morus alba* Wood Log P: 3.38	PO	-	-	[51]
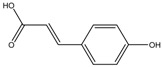 p-Coumaric acid	Formula: C_9_H_8_O_3_ MW: 164.05 Origin: Plant Log P: 1.54	~40% (100 µM)	Pigment eye	-	[43,69]
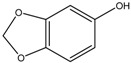 Sesamol	Formula: C_7_H_6_O_3_ MW: 138.12 Origin: *Sesamum indicum* Log P: 1.42	~55% (50 µM)	~30% (50 µM)	~20–30% (50 µM)	[63]
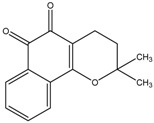 β-lapachone	Formula: C_15_H_14_O_3_ MW: 242.27 Origin: *Tabebuia avellandedae* Log P: 1.68	PO	~70% (3.2 µM)	~40% (3.2 µM)	[64]
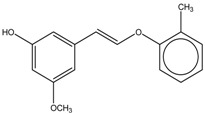 2-Methylphenyl-E-(3-hydroxy-5-methoxy)-styryl ether	Formula: C_16_H_16_O_3_ MW: 256.11 Origin: Synthetic Log P: 4.02	PO	-	-	[41]
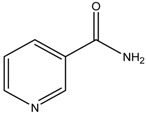 Niacinamide	Formula: C_6_H_6_ON_2_ MW: 122.12 Origin: Plant Log P: −0.35	PO	-	-	[41]
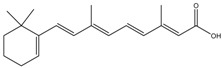 Tretinoin	Formula: C_19_H_26_O_2_ MW: 286.41 Origin: Natural/Synthetic Log P: 4.47	PO	-	-	[41]
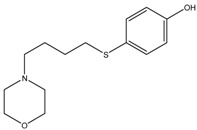 2-Morpholinobutyl)-4-thiophenol	Formula: C_14_H_21_O_2_NS MW: 267.39 Origin: Plant Log P: 2.24	PO	-	-	[41]

Note: MW, molecular weight (g/mol); ICp, phenotype pigmentation level; ICm, melanin content level; ICt, tyrosinase activity level; PO, Phenotype observation. ICp, ICm and ICt in percentage (%) as compared to untreated control.

**Table 2:** The bioactive compounds (intermediate molecular weight, 300-500 g/mol) and their antimelanogenic activity.

Bioactive Compounds	Description	ICp	ICm	ICt	References
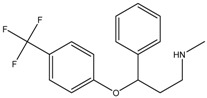 Fluoxetine	Formula: C_17_H_18_F_3_NO MW: 309.33 Origin: Synthetic Log P: 4.27	PO	~50% (10 µM)	~80% (10 µM)	[46]
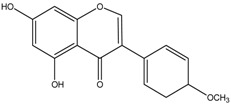 Biochanin A	Formula: C_17_H_18_O_5_ MW:302.12 Origin: *Trifolium pratense* Log P: 0.92	-	~50% (176 µM)	~40% (176 µM)	[3]
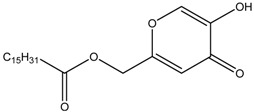 Kojic acid palmitate	Formula: C_22_H_36_O_5_ MW:380.52 Origin: Synthetic Log P: 3.86	-	~40% (62.5 ug/mL)	~37% (62.5 ug/mL)	[40]
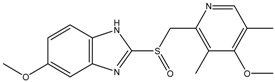 Omeprazole	Formula: C_17_H_19_N_3_O_3_S MW: 345.42 Origin: Synthetic Log P: 2.17	63% (60 µM)	~60% (60 µM)	~50% (60 µM)	[53]
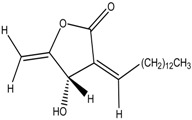 Linderanolide B	Formula: C_19_H_32_O_3_ MW: 308.46 Origin: *Cinnamomum subavenium* Log P: 4.91	PO	-	-	[71,72,73]
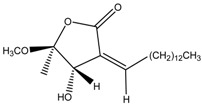 Subamolide A	Formula: C_20_H_36_O_4_ MW: 340.50 Origin: *Cinnamomum subavenium* Log P: 5.68	PO	-	-	[71,72,73]
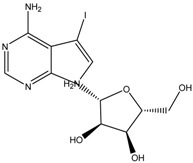 5-iodotubercidin	Formula: C_13_H_21_IN_4_O_4_ MW: 424.23 Origin: Marine red alga Log P: −0.75	PO	-	-	[74]
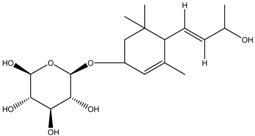 Picrionoside A	Formula: C_18_H_30_O_7_ MW:358.43 Origin: Ginseng Log P: 0.89	PO	-	-	[75]
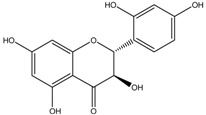 Trans-Dihydromorin	Formula: C_15_H_12_O_7_ MW: 304.25 Origin: *Morus alba Wood* Log P: 0.58	PO	-	-	[51]
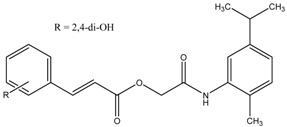 Compound 6d	Formula: C_21_H_24_NO_4_ MW:344.41 Origin: Synthetic Log P: ~4	~40–55%, 50 µM	-	-	[52]
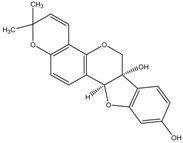 (i.e., Glyceollin I)	Formula: C_20_H_18_O_5_ MW:338.35 Origin: Soybean Log P: 2.53	PO	~30% (10 µM)	~60% (10 µM)	[76]
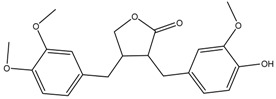 Arctigenin	Formula: C_21_H_24_O_6_ MW:372.41 Origin: *Fructus Arctii* Log P: 3.51	PO	-	-	[58]
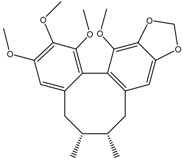 Gomisin N	Formula: C_23_H_28_O_6_ MW:400.46 Origin: *Schisandra chinensis* Log P: 4.95	PO	40% (30 µM)	-	[54]
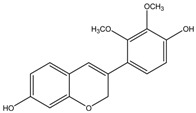 Haginin A	Formula: C_17_H_16_O_5_ MW:300.31 Origin: *Lespedeza cyrtobotrya* Log P: 2.45	PO	-	~55% (4 µM)	[77]
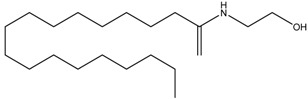 Oleoylethanolamide	Formula: C_21_H_43_NO MW:325.57 Origin: Intestine Log P: 6.31	67% (100 µM) 49.5% (150 µM)	-	-	[78]
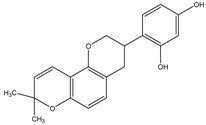 Glabridin	Formula: C_20_H_20_O_4_ MW:324.37 Origin: *Glycyrrhiza glabra* Log P: 3.73	No effect	-	-	[62]
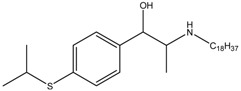 Suloctidil	Formula: C_30_H_55_NOS MW:477.83 Origin: Synthetic Log P: 9.56	PO	-	-	[79]
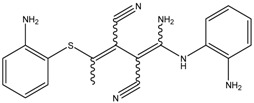 MEK-1	Formula: C_19_H_18_N_6_S MW:362.45 Origin: Synthetic Log P: 1.48	PO	-	-	[41]

Note: MW, molecular weight (g/mol); ICp, phenotype pigmentation level; ICm, melanin content level; ICt, tyrosinase activity level; PO, Phenotype observation. ICp, ICm and ICt in percentage (%) as compared to untreated control.

**Table 3:** The bioactive compounds (high molecular weight, 500–1000 g/mol) and their antimelanogenic activity.

Bioactive Compounds.	Description	ICp	ICm	ICt	References
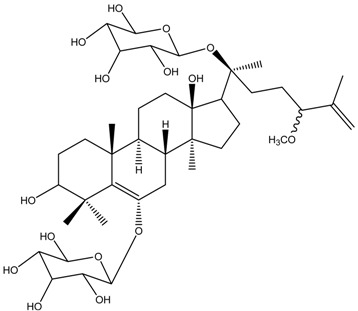 Floralginsenoside A	Formula: C_40_H_66_O_15_ MW: 786.44 Origin: Panax ginseng Log P:-	PO	82% (80 µM) 79% (160 µM)	83% (80 µM) 78% (160 µM)	[42]
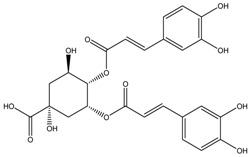 4,5-O-Dicaffeoylquinic Acid	Formula: C_25_H_24_O_12_ MW:516.13 Origin: *Artemisia capillaris Thunberg* (Oriental Wormwood) Log P: 0.49	PO	50% (75 ug/mL)	60% (75 ug/mL)	[31]
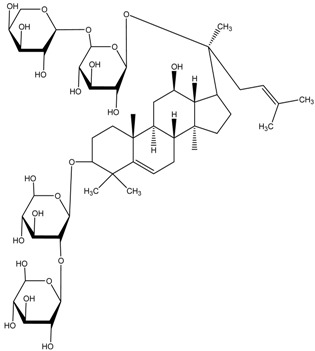 Ginsenoside Rb2	Formula: C_48_H_78_O_22_ MW: 1007.12 Origin: Panax ginseng Log P:-	PO	80% (80 µM)	78% (80 µM)	[39]
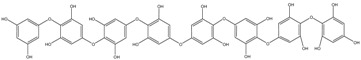 Octaphlorethol A	Formula: C_48_H_34_O_24_ MW: 994.77 Origin: *Ishige foliacea* Log P:-	PO	~75% (25 µM)	~67% (25 µM)	[84]

Note: MW, molecular weight (g/mol); ICp, phenotype pigmentation level; ICm, melanin content level; ICt, tyrosinase activity level; PO, Phenotype observation. ICp, ICm and ICt in percentage (%) as compared to untreated control.

**Table 4:** The bioactive compounds, crude and formulation and their antimelanogenic activity.

Bioactive Compounds/Crude/Formulation	Description	ICp	ICm	ICt	References
Herbal prescription LASAP-C	Origin: four herbal medicines-*Rehmanniae Radix Crudus, Lycii Fructus*, *Scutellariae Radix*, *Angelicae Dahuricae Radix*	PO	-	-	[87]
*Salix alba* bark extract	Origin: *Salix alba*	-	40% 400 ug/mL	-	[85]
(+)-Dehydrovomifoliol, (6R,7E,9R)-9-hydroxy-4,7-megastigmadien-3-one, (3S,5R,8R)-3,5-dihydroxymegastigma-6,7-dien-9-one, roseoside, and citroside A	Origin: Silkworm (*Bombyx mori* L.) dropping	PO	-	-	[7]
*Magnolia officalis* extract	Origin: *Magnolia officalis*	PO	Melanin 70–80%, 6.25 ug/mL	60–70%, 6.25 ug/mL	[67]
*Ganoderma formosanum* mycelium extract	Origin: *Ganoderma formosanum*	-	50% (400 ppm)	50%, (400 ppm)	[44,90]
Flavonoid	Origin: *Blumea balsamifera* L.	-	42% (300 ug/mL)	-	[66]
Marine Pseudomonas Extract	Origin: *Pseudomonas sp*	PO	-	-	[88]
*Anoectochilus* extract	Origin: *Anoectochilus*	PO	-	-	[86]
*Anoectochilus roxburghii* extract	Origin: *Anoectochilus roxburghii*	PO	-	-	[56]
Alcohol extracts of *Narcissus* bulb	Origin: *Narcissus*	PO	-	-	[89]
Ethanol Extract of *Discorea* *nipponica* Makino	Origin: *Discorea nipponica*	PO	50% (25 ug/mL)	-	[91]

Note: MW, molecular weight (g/mol); ICp, phenotype pigmentation level; ICm, melanin content level; ICt, tyrosinase activity level; PO, Phenotype observation. ICp, ICm and ICt in percentage (%) as compared to untreated control.
